# Echinochrome A Inhibits Melanogenesis in B16F10 Cells by Downregulating CREB Signaling

**DOI:** 10.3390/md20090555

**Published:** 2022-08-29

**Authors:** Mi Ran Choi, Heejin Lee, Hyoung Kyu Kim, Jin Han, Jung Eun Seol, Elena A. Vasileva, Natalia P. Mishchenko, Sergey A. Fedoreyev, Valentin A. Stonik, Won Seok Ju, Dai-Jin Kim, Sang-Rae Lee

**Affiliations:** 1 Department of Pharmacology, Ajou University School of Medicine, Suwon 16499, Korea; 2 Laboratory Animal Research Center, Ajou University School of Medicine, Suwon 16499, Korea; 3 Department of Psychiatry, Seoul St. Mary’s Hospital, College of Medicine, The Catholic University of Korea, Seoul 06591, Korea; 4 Department of Physiology, College of Medicine, Cardiovascular and Metabolic Disease Center, Smart Marine Therapeutic Center, Department of Health Sciences and Technology, Graduate School, Inje University Busan, Busan 50834, Korea; 5 Department of Dermatology, Inje University Busan Paik Hospital, Inje University College of Medicine, Busan 47392, Korea; 6 G.B. Elyakov Pacific Institute of Bioorganic Chemistry, Far-Eastern Branch of the Russian Academy of Science, 690022 Vladivostok, Russia

**Keywords:** echinochrome A, melanin, α-MSH, pCREB, skin whitening

## Abstract

Excessive increase in melanin pigment in the skin can be caused by a variety of environmental factors, including UV radiation, and can result in spots, freckles, and skin cancer. Therefore, it is important to develop functional whitening cosmetic reagents that regulate melanogenesis. In this study, we investigated the effects of echinochrome A (Ech A) on melanogenesis in the B16F10 murine melanoma cell line. We triggered B16F10 cells using α-MSH under Ech A treatment to observe melanin synthesis and analyze expression changes in melanogenesis-related enzymes (tyrosinase, tyrosinase-related protein 1 (TYRP1), and tyrosinase-related protein 2 (TYRP2)) at the mRNA and protein levels. Furthermore, we measured expression changes in the microphthalmia-associated transcription factor (MITF), CREB, and pCREB proteins. Melanin synthesis in the cells stimulated by α-MSH was significantly reduced by Ech A. The expression of the tyrosinase, TYRP1, and TYRP2 mRNA and proteins was significantly decreased by Ech A, as was that of the MITF, CREB, and pCREB proteins. These results show that Ech A suppresses melanin synthesis by regulating melanogenesis-related enzymes through the CREB signaling pathway and suggest the potential of Ech A as a functional agent to prevent pigmentation and promote skin whitening.

## 1. Introduction

Melanin pigmentation is caused by cosmetological and medical factors such as sunlight, hormonal changes, inflammation, age, skin injuries, or drugs [1,2,3,4]. If skin is exposed to sunlight, the main environmental factor regulating melanin pigmentation, melanin, absorbs the UV light contained in the sunlight to protect the skin below the dermis from UV damage and harmful factors such as reactive oxygen species and free radicals generated in the skin [5,6]. However, if melanin is excessively synthesized or pigmented as a result of these factors, spots, freckles, and even skin cancer can occur [1,2,3,4]. To prevent pigmentation and obtain a whitening effect, it is necessary to reduce the production of melanin by inhibiting the production process. 

Melanin is synthesized in the melanosomes of melanocytes in the epidermis and determines skin and hair color [7]. The melanin synthesis process is regulated by a series of molecular and enzymatic reactions involving the microphthalmia-associated transcription factor (MITF) and three enzymes (tyrosinase, tyrosinase-related protein 1 (TYRP1), and tyrosinase-related protein 2 (TYRP2)) that are transcriptionally controlled by MITF in melanocytes [8,9,10]. Tyrosinase is an essential and rate-limiting enzyme involved in the initial step of the melanogenic pathway. It catalyzes the conversion of L-tyrosine to dopaquinone and of 3,4-dihydroxyphenylalanine (L-DOPA) to dopaquinone in the Raper–Mason pathway [11]. Sometimes, L-DOPA is produced by oxidation of L-tyrosine to activate tyrosinase. If dopaquinone is not converted to dopachrome by an additional enzymatic reaction, it enters the pheomelanin (one of the two main types of melanin) synthesis pathway. On the other hand, dopachrome converted from dopaquinone is entered into the eumelanin (the other type of melanin) synthesis pathway by TYRP1 and TYRP2 [11,12]. Because of the direct involvement of these proteins in melanin synthesis, tyrosinase, TYRP1, TYRP2, and MITF are attracting attention as candidates for the development of skin-whitening cosmetics. 

When epidermal skin is exposed to exogenous stimuli, including UV, keratinocytes secrete the α-melanocyte-stimulating hormone (α-MSH). The secreted α-MSH binds to the melanocortin 1 receptor (MC1R) on the melanocyte surface to stimulate serial activation of upstream mediators in the CREB signaling pathway by inducing the activation of adenylate cyclase [13]. Eventually, phosphorylated CREB (pCREB), the active form of CREB, translocates into the nucleus and promotes the transcription of MITF, which induces the expression of tyrosinase, TYRP1, and TYRP2 to increase melanin synthesis [14,15]. 

**Figure 1:**
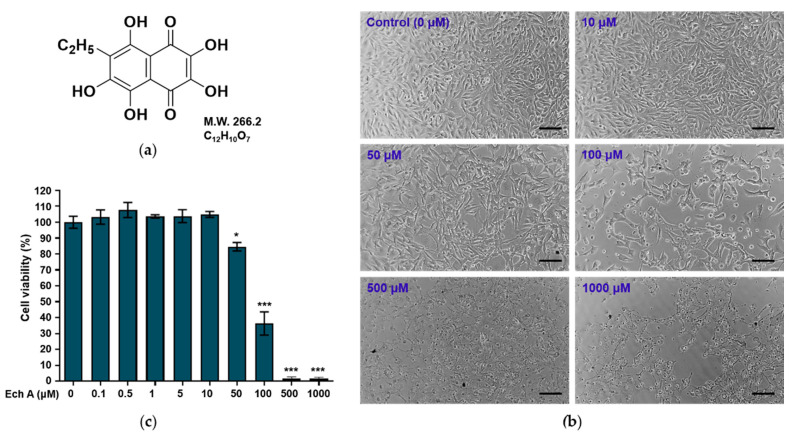
Effects of Ech A on the viability of mouse melanoma B16F10 cells. (**a**) Chemical structure of Ech A. (**b**) Morphology of the cells exposed to different concentrations of Ech A for 24 h. Scale bar = 100 μm. (**c**) Viability of the cells exposed to Ech A. The cells were treated with different concentrations (0, 0.1, 0.5, 1, 10, 50, 100, 500, and 1000 μM) of Ech A for 24 h, and cell viability was measured using a Cell Counting Kit-8. Ech A treatments were conducted in three independent replicates to guarantee reliable results. To compare differences in cell viability between the control (0 μM) and Ech A-treated cells, one-way ANOVA was used. Note: * significantly different from the control cells (* *p* < 0.05 and *** *p* < 0.0001).

Echinochrome A (Ech A) is a natural pigment commonly found in sea urchins; its chemical structure is 6-ethyl-2,3,5,7,8-pentahydroxy-1,4-naphthoquinone (Figure 1a) [16]. Ech A has antioxidant, anti-inflammatory, antiviral, and chelating capabilities [17,18,19,20], is used as a therapeutic agent for a few diseases, and has been evaluated as a therapeutic candidate in in vitro and in vivo disease models [20,21]. Its water-soluble sodium salt form has been used to treat myocardial ischemia/reperfusion injury and degenerative diseases of the retina and cornea [22]. Previous studies have shown that Ech A alleviates not only inflammation and fibrosis in scleroderma due to bleomycin [23], but also atopic dermatitis-like skin lesions in NC/Nga mice [21], implying that Ech A could be a reagent for healing skin diseases. 

Therefore, we hypothesized that Ech A would suppress melanogenesis and be a good candidate for preventing pigmentation and enhancing skin whitening. If Ech A affects melanogenesis as we hypothesize, it could be developed into a skin-whitening cosmetic based on a natural marine product. The B16F10 murine melanoma cell line is widely used to analyze melanogenesis-related mechanisms and develop whitening cosmetics [24,25,26]. In this study, we identified the concentration of Ech A that causes cytotoxicity in B16F10 cells and examined whether Ech A inhibits melanin biosynthesis. Furthermore, we investigated how Ech A affects the parts of melanin biosynthesis that are regulated by the CREB signaling pathway. 

## 2. Results

### 2.1. Effects of Ech A on Cell Viability

To investigate the effects of Ech A on the viability of B16F10 cells, we treated the cells with different concentrations of Ech A for 24 h. The morphology and viability of the cells exposed to 0.1–10 μM Ech A did not differ from those of the control cells (Figure 1b,c). Treatment with 50 μM Ech A induced altered morphology in the cells and a significant (*p* < 0.05) decrease in cell viability. Exposure to 100 μM Ech A reduced cell viability by more than 50%, indicating that that concentration is cytotoxic to B16F10 cells.

### 2.2. Ech A Inhibits L-DOPA Oxidation

To evaluate whether Ech A inhibits tyrosinase activity and L-DOPA oxidation in vitro, arbutin was used as the positive control [27,28]. In this study, the half-maximal inhibitory concentration (IC_50_) values of arbutin for inhibiting tyrosinase activity and L-DOPA oxidation were 126 ± 23.11 μM and 3279.74 ± 17.14 μM, respectively (Table S1). Based on those results, we selected 72 and 720 μM arbutin to test tyrosinase inhibition and 900 and 3670 μM arbutin to test L-DOPA oxidation inhibition as the positive controls. 

**Figure 2:**
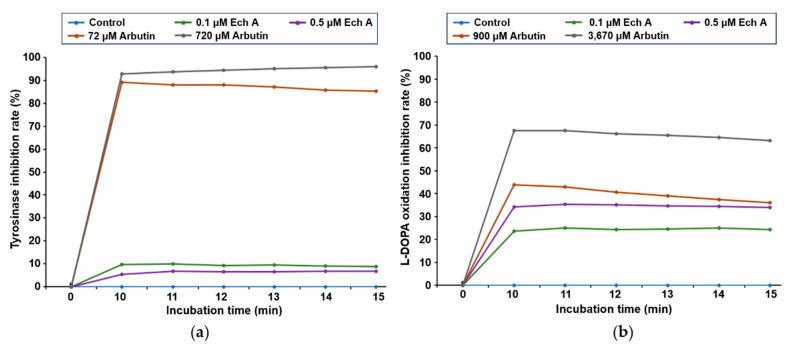
Inhibitory effects of Ech A on tyrosinase activity and L-DOPA oxidation. (**a**) In vitro tyrosinase inhibition assay. The inhibitory effect of Ech A on tyrosinase activity was measured in a time-dependent manner. (**b**) In vitro L-DOPA oxidation inhibition assay. The inhibitory effect of Ech A on the oxidation of L-DOPA to dopaquinone was measured in a time-dependent manner. Arbutin was used as the positive control.

When we measured how well Ech A exposure at different concentrations and times inhibited tyrosinase activity and L-DOPA oxidation, we found that Ech A barely inhibited tyrosinase activity at 0.1 and 0.5 μM, whereas arbutin inhibited tyrosinase activity in a concentration-dependent manner (Figure 2a). L-DOPA oxidation was slightly inhibited by low concentrations (0.1 and 0.5 μM) of Ech A (Figure 2b). We could not detect inhibition of tyrosinase activity or L-DOPA oxidation at Ech A concentrations higher than 0.5 μM because of the disturbance in the OD measurement caused by the brown color of the solution.

### 2.3. Ech A Inhibits mRNA of Tyrosinase in B16F10 Melanoma Cells

To evaluate how well Ech A inhibited the expression of the *tyrosinase*, *Tyrp1*, and *Tyrp2* genes, kojic acid was used as the positive control [29,30]. When we exposed B16F10 cells to serial concentrations (0, 12.5, 25, 50, 100, and 200 μM) of kojic acid, 12.5–50 μM kojic acid did not affect cell viability, but 100 and 200 μM kojic acid significantly decreased it (Figure S1). Therefore, 50 μM kojic acid was selected as the positive control. 

**Figure 3:**
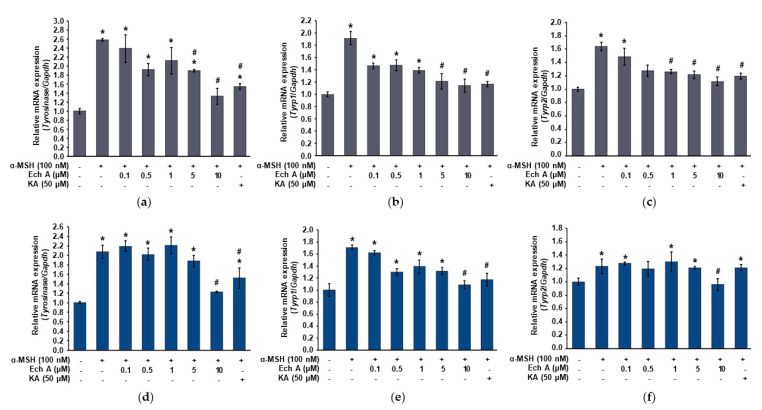
Expression of the *tyrosinase*, *Tyrp1*, and *Tyrp2* mRNA in the B16F10 cells exposed to Ech A. Ech A or kojic acid (KA, positive control) was administered to the cells 1 h before α-MSH treatment, and then the cells were incubated for 24 and 48 h. The expression of the *tyrosinase*, *Tyrp1*, and *Tyrp2* mRNA was analyzed using qRT-PCR after extracting total RNA. (**a**–**c**) Expression changes of the *tyrosinase*, *Tyrp1*, and *Tyrp2* mRNA in the cells exposed to Ech A or KA for 24 h. (**d**–**f**) Expression changes of the *tyrosinase*, *Tyrp1*, and *Tyrp2* mRNA in the cells exposed to Ech A or KA for 48 h. Note: * significantly different from the control cells (*p* < 0.05); ^#^ significantly different from the cells exposed only to α-MSH (*p* < 0.05).

To investigate whether Ech A affects the expression of the *tyrosinase*, *Tyrp1*, and *Tyrp2* mRNA, we used quantitative reverse transcription PCR (qRT-PCR) to measure the expression of those genes in the B16F10 cells exposed to different concentrations (0, 0.1, 0.5, 1, 5, and 10 μM) of Ech A, 100 nM α-MSH, or 50 μM kojic acid. *Tyrosinase*, *Tyrp1*, and *Tyrp2* were significantly increased after both 24 and 48 h of exposure to α-MSH (Figure 3). On the other hand, although the expression patterns of *tyrosinase*, *Tyrp1*, and *Tyrp2* differed at some concentrations of Ech A between 24 and 48 h of exposure, the cells exposed to α-MSH and 10 μM Ech A together showed a significant decrease in *tyrosinase*, *Tyrp1*, and *Tyrp2* expression at both 24 and 48 h compared with the cells exposed to only α-MSH. Therefore, we believe that 10 μM Ech A efficiently inhibits the increased expression of those genes caused by α-MSH.

### 2.4. Ech A Suppresses Melanin Production

**Figure 4:**
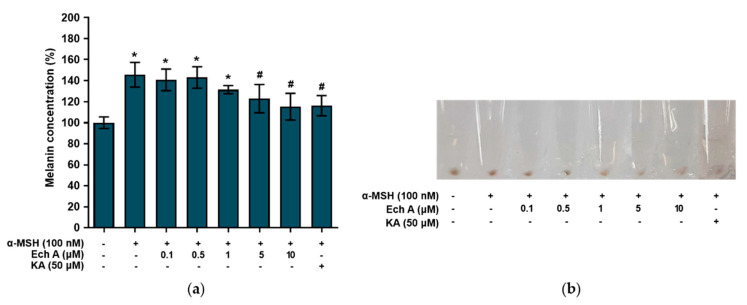
Effects of Ech A on melanin synthesis in B16F10 cells. Ech A or kojic acid (KA) was administered to the cells 1 h before α-MSH treatment, and then the cells were incubated for 48 h. (**a**) Melanin content in B16F10 cells. Note: * significantly different from the control cells (*p* < 0.05); ^#^ significantly different from the cells exposed only to α-MSH (*p* < 0.05). (**b**) Images of cell pellets.

To investigate the effects of Ech A on melanin synthesis, we measured the melanin concentration in the B16F10 cells that had been exposed to different concentrations of Ech A, α-MSH (100 nM), or kojic acid (50 μM). The cells exposed to α-MSH alone and α-MSH + Ech A (0.1, 0.5, and 1 μM) showed a significant increase in melanin concentration compared with the control cells (Figure 4a), implying that these concentrations of Ech A did not inhibit the melanin synthesis stimulated by α-MSH. On the other hand, the α-MSH + Ech A (5 and 10 μM) treatments induced a significant decrease in melanin synthesis in B16F10 cells compared with the cells exposed to α-MSH alone. In fact, 10 μM Ech A exhibited the same melanin production inhibitory effect as kojic acid, a direct inhibitor of tyrosinase. Furthermore, when the cells were treated with different concentrations of Ech A, the brown color of the cell pellets blurred due to the decrease in melanin production as the concentration of Ech A increased (Figure 4b). 

### 2.5. Ech A Inhibits Melanin Synthesis through the CREB Signaling Pathway

**Figure 5:**
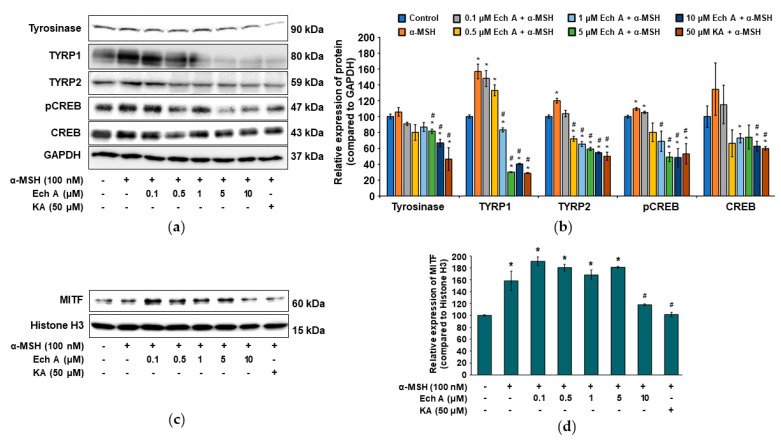
Effects of Ech A on the expression of melanogenesis-related proteins in B16F10 cells. Ech A or kojic acid (KA) was administered to the cells 1 h before α-MSH treatment, and then the cells were incubated for 48 h. (**a**) Immunoblots of the tyrosinase, TYRP1, TYRP2, pCREB, and CREB proteins. (**b**) Densitometric analysis of the relative intensity of the proteins detected in (**a**). (**c**) Immunoblot of the nuclear MITF protein. (**d**) Densitometric analysis showing the relative intensity of the nuclear MITF protein compared with the nuclear histone H3 protein. Note: * significantly different from the control cells (**b**,**d**) (*p* < 0.05); ^#^ significantly different from the cells exposed only to α-MSH (**b**,**d**) (*p* < 0.05).

We investigated the expression of the proteins related to melanin synthesis and the CREB signaling pathway after exposing B16F10 cells to Ech A, α-MSH, or kojic acid. The TYRP1 and TYRP2 proteins were significantly increased by α-MSH treatment alone, and that increase was reversed by cotreatment with Ech A, showing that Ech A decreased protein production, especially of TYRP2, in a dose-dependent manner (Figure 5a,b). Tyrosinase was also decreased significantly by Ech A, with the highest decrease after exposure to 10 μM Ech A. In addition, pCREB expression in the cells exposed to both α-MSH and Ech A (5 or 10 μM) was significantly lower than in the cells exposed to only α-MSH and the control cells and similar to the level observed in the cells exposed to both α-MSH and 50 μM kojic acid (Figure 5a,b). Furthermore, the total CREB expression was significantly lower in the cells exposed to both α-MSH and 10 μM Ech A than it was in those exposed to only α-MSH and in the control cells. Because we could not detect the expression of MITF in whole cells, we measured its expression in the nucleus. The cells exposed to α-MSH and Ech A (0.1, 0.5, 1, or 5 μM) together showed a higher MITF expression than those exposed only to α-MSH and the control cells, whereas treatment with both 10 μM Ech A and α-MSH induced a decrease in MITF expression in the nucleus (Figure 5c,d). Therefore, we believe that Ech A inhibits melanin synthesis through the CREB signaling pathway.

## 3. Discussion

Melanin absorbs UV light from sunlight to protect the skin from UV damage, and it scavenges reactive oxygen species by absorbing harmful external factors. Thus, exposing skin to UV augments melanin production and pigmentation. Synthesis and pigmentation of excessive melanin cause spots, freckles and even skin cancer. Because people use functional cosmetics to whiten their skin, the interest in the study of melanogenesis and development of whitening cosmetics is growing. Based on previous observations that Ech A not only has antioxidative and anti-inflammatory effects, but also suppresses UVB-induced photoaging [16,31,32], we investigated the effects of Ech A on melanogenesis in melanocytes and examined the potential of Ech A as a whitening cosmetic ingredient. 

When we evaluated the cytotoxicity of Ech A in B16F10 cells, we found that Ech A did not cause cytotoxicity at up to 10 μM, but 50 and 100 μM Ech A decreased cell viability. Some previous studies reported that cytotoxicity was induced at concentrations different from those in this study [20,33]. One study reported that Ech A did not affect the viability of mouse embryonic stem cells at up to 500 μM and that up to 50 μM Ech A enhanced cardiomyocyte differentiation from embryonic bodies [33]. Another study reported that exposure to 0.5–10 μM Ech A significantly increased cell viability in human cardiac progenitor cells, and 50 μM Ech A had no effect on cell viability [20]. However, above 100 μM, Ech A significantly decreased cell viability. On the basis of previous studies and our results, we think that the concentration of Ech A that affects cell viability differs by cell type.

Melanin is mainly synthesized in two types, pheomelanin and eumelanin [11,12]. Pheomelanin varies from yellow to reddish-brown color, while eumelanin varies from light brown to black color. Tyrosinase, the rate-limiting enzyme in melanogenesis, participates in the early stages (conversion of L-tyrosine to dopaquinone and oxidation of L-DOPA to dopaquinone) of melanin synthesis [11]. In this study, in vitro tyrosinase activity and L-DOPA oxidation were evaluated to validate the potential of Ech A as an anti-melanogenic agent. Compared with the inhibition of tyrosinase activity and L-DOPA oxidation caused by arbutin, Ech A showed minimal inhibitory effects. The Ech A solution is dark brown, so we could not detect signals at concentrations higher than 0.5 μM Ech A. Therefore, it is necessary to use other experimental methods to further identify whether Ech A regulates melanin synthesis.

Arbutin, which we used as the positive control, is a cosmetic ingredient used for whitening by inhibition of tyrosinase [34,35]. However, its use is limited because of decomposition or discoloration due to its poor stability, occurrence of an off-flavor, unclear side effects, and safety issues. Therefore, a whitening agent that directly scavenges tyrosinase and inhibits the formation of melanogenesis-related enzymes at the transcription and translation levels is needed. TYRP1 and TYRP2 regulate eumelanin synthesis [11,12]. Therefore, we investigated whether Ech A affects the expression of the *tyrosinase*, *Tyrp1*, and *Tyrp2* mRNA and found that the expression of those genes triggered by α-MSH was decreased after both 24 and 48 h of exposure to Ech A. In particular, treatment with 10 μM Ech A reduced the expression of the three genes to the levels expressed in the control cells. Taken together, our results show that Ech A controls melanogenesis by inhibiting the gene expression of three enzymes (tyrosinase, TYRP1, TYRP2). In this study, 5 and 10 μM Ech A inhibited α-MSH-stimulated melanin synthesis in B16F10 cells. In particular, the melanin concentration in the cells exposed to 10 μM Ech A was equal to the melanin concentration in the cells exposed to 50 μM kojic acid, the control inhibitor of melanin synthesis. Therefore, our results suggest that Ech A can inhibit melanin synthesis at a lower concentration than kojic acid. 

Several signaling pathways, including CREB, are involved in melanin synthesis [28]. Keratinocytes exposed to UV secrete α-MSH, which binds MC1R on the melanocyte surface. The α-MSH/MC1R complex triggers intracellular cAMP production, which activates the CREB, JNK, MAPK/ERK, and p38 MAPK pathways [28,36,37,38]. These signaling pathways promote MITF expression and activation, followed by increased expression of downstream proteins (tyrosinase, TYRP1, and TYRP2) and increased melanin synthesis. In this study, Ech A significantly inhibited the expression of the pCREB and CREB stimulated by α-MSH. In fact, the expression of CREB and pCREB in the cells treated with 10 μM Ech A was lower than that in the control cells. The expression of the tyrosinase, TYRP1, and TYRP2 proteins stimulated by α-MSH was also inhibited by Ech A, and 5 and 10 μM Ech A suppressed their expression with and without α-MSH stimulation. When we measured the amount of the MITF protein in the nucleus (because MITF is an important nuclear transcription factor modulating melanin synthesis [39]), we found that 10 μM Ech A effectively prevented the expression of MITF triggered by α-MSH. However, cotreatment with 0.1 μM Ech A and α-MSH tended to increase the expression of MITF compared with the cells exposed to α-MSH alone. Because 10 μM Ech A alone significantly reduced MITF expression, we think that either a low concentration of Ech A could not inhibit α-MSH stimulation or that it was an experimental error. In addition to Ech A, other natural products obtained from marine animals and plants have been investigated as potential skin-whitening agents [40,41]. Chen et al. [40] found that 196 mM chitosan from crustaceans inhibited the expression of the MITF, tyrosinase, TYRP1, and TYRP2 proteins and the tyrosinase activity triggered by α-MSH in B16F10 cells, so they suggested chitosan as a potential skin-whitening agent. Ech A is also a marine product that inhibits the expression of the MITF, tyrosinase, TYRP1, and TYRP2 proteins and the tyrosinase activity triggered by α-MSH at a concentration of 10 μM; therefore, we argue that Ech A would be more effective than chitosan for suppressing melanin synthesis. Taken together, our results suggest that Ech A is a strong inhibitor of melanogenesis.

## 4. Materials and Methods

### 4.1. Preparation of Ech A

Histochrome^®^ containing 1% Ech A (6-ethyl-2,3,5,7,8-pentahydroxy-1,4-naphthoquinone, pharmaceutical, state registration number PN002362/01–2003) was provided by G.B. Elyakov Pacific Institute of Bioorganic Chemistry, FEB, RAS, Russia. Histochrome^®^ composition is 1% Ech A in a 0.9% isotonic solution (sodium carbonate and sodium chloride, 37.5 mM).

### 4.2. Cell Culture

The B16F10 murine melanoma cell line was purchased from the Korean Cell Line Bank (KCLB) (Seoul, Korea). The cells were cultured in Dulbecco’s modified Eagle’s medium with 10% FBS and 100 units/ml penicillin plus 100 mg/ml streptomycin at 37 °C with 5% CO_2_ according to the KCLB guideline. After the cells reached 70–80% confluence, they were subcultured using 0.25% trypsin/EDTA (Thermo Fisher Scientific, Waltham, MA, USA).

### 4.3. Cell Cytotoxicity Assay

To investigate the cytotoxicity of Ech A, cell viability was assayed using a Cell Counting Kit-8 (CCK-8; Dojindo, Kumamoto, Japan) according to the manufacturer’s instructions. Prior to Ech A treatment, B16F10 cells were seeded in a 96-well plate at a density of 10,000 cells per well and incubated at 37 °C and 5% CO_2_ for 24 h. Then, the cells were exposed to a fresh serum-free medium with or without different concentrations of Ech A and incubated at 37 °C and 5% CO_2_ for another 24 h. At that time, CCK-8 solution was added to the plate containing the cells, and the plate was incubated at 37 °C for 2 h. Absorbance was measured at 450 nm using a microplate reader (Power Wave XS2, BioTeck, Winooski, VT, USA).

### 4.4. Inhibition of Tyrosinase Activation and L-DOPA Oxidation

The in vitro tyrosinase inhibition assay was performed using previously described methods [42]. For the reaction mixture, 120 μL of PBS (0.1 M, pH 6.5), 20 μL of serially diluted Ech A (0, 0.1, 0.5, 1, 5, 10, and 50 μM), 20 μL of mushroom tyrosinase (2000 U/mL) (Sigma-Aldrich, St. Louis, MO, USA), and 40 μL of L-tyrosine (1.5 mM) (Sigma-Aldrich, St. Louis, MO, USA) were added to a 96-well plate that was then incubated at 37 °C for 10–15 min, when the optical density of the wells was measured at 490 nm. The concentration of Ech A at which half the original tyrosinase activity was inhibited (IC_50_) was determined using two concentrations (72 and 720 μM) of arbutin (Sigma-Aldrich, St. Louis, MO, USA) as the positive controls. 

The in vitro L-DOPA oxidation inhibition assay was also performed using previously described methods [43]. For the reaction mixture, 150 μL of PBS (0.1 M, pH 6.5), 20 μL of serially diluted Ech A (0, 0.1, 0.5, 1, 5, 10, and 50 μM), 20 μL of mushroom tyrosinase (2000 U/mL) (Sigma-Aldrich, St. Louis, MO, USA), and 10 μL of L-DOPA (60 μM) (Sigma-Aldrich, St. Louis, MO, USA) were added to a 96-well plate that was then incubated at 37 °C for 10–15 min, when the optical density of the wells was measured at 475 nm. The concentration of Ech A at which half the original L-DOPA oxidation was inhibited (IC_50_) was determined using two concentrations (900 and 3670 μM) of arbutin as the positive controls. 

### 4.5. Melanin Synthesis Assay

B16F10 cells were seeded in a 6-well plate at a density of 50,000 cells per well and incubated at 37 °C and 5% CO_2_ for 24 h. Then, the culture medium was exchanged with a fresh phenol red-free growth medium containing Ech A (0, 0.1, 0.5, 1, 5, and 10 μM) or 50 μM kojic acid and the plate was incubated again at 37 °C and 5% CO_2_. After 1 h of incubation, 100 nM α-MSH (Sigma-Aldrich, St. Louis, MO, USA) was added, and the plate was incubated at 37 °C and 5% CO_2_ for an additional 47 h. The cultured cells were harvested, counted, and lysed with 1 N NaOH containing 10% dimethyl sulfoxide at 80 °C for 10 min. The cell lysate was centrifuged at 13,000× *g* for 2 min, and the total protein concentration of the supernatant was measured. The supernatant was transferred to a 96-well plate, and the amount of melanin was measured at 490 nm. The melanin content of the total protein was calculated per test condition and is presented as a percentage of the control.

### 4.6. Quantitative RT-PCR

B16F10 cells were incubated with a growth medium containing Ech A (0, 0.1, 0.5, 1, 5, and 10 μM) or 50 μM kojic acid at 37 °C and 5% CO_2_ for 1 h. Then, 100 nM α-MSH was added, and the cells were incubated at 37 °C and 5% CO_2_ for an additional 23 h or 47 h. To analyze the expression of the *Tyr*, *Tyrp1*, *Tyrp2*, and *Mitf* mRNA, we performed qRT-PCR. The cells exposed to Ech A or kojic acid for 24 h and 48 h were harvested, and total RNA was extracted using a Trizol reagent (Thermo Fisher Scientific, Waltham, MA, USA). The extracted total RNA was reverse-transcribed with a TOPscript^TM^ cDNA Synthesis Kit (Enzynomics, Seoul, Korea). For quantitative PCR, 5 µL of TB Green Premix Ex Taq (Takara Bio, Otsu, Japan), 5 pmol of the forward primer, 5 pmol of the reverse primer, and 1 µL of cDNA were added with water to a final volume of 25 µL. The mixture was amplified for 35 cycles using a CFX96 Touch Real-Time PCR Detection System (Bio-Rad, Hercules, CA, USA). The cycle number at which a statistically significant increase in each gene was first detected (threshold cycle, Ct) was normalized to the Ct for *Gapdh*. The relative expression differences between the genes were calculated using the 2^−ΔΔCT^ method [44]. The primers used for gene amplification are presented in Table 1.

### 4.7. Western Blot Analysis

The cells cultured under the same conditions as in the melanin synthesis assay were lysed with a PRO-PREP protein extraction solution (iNtRON Biotechnology, Gyeonggi, Korea) containing protease and phosphatase inhibitors (Quartett, Berlin, Germany) and incubated at 4 °C for 20 min. The cell lysates were centrifuged at 13,000× *g* and 4 °C for 5 min, and the supernatants were transferred into fresh tubes. The extracted protein concentration was measured using a Bio-Rad Protein Assay Kit (Bio-Rad, Hercules, CA, USA) according to the manufacturer’s instructions. The proteins were denatured by heating, separated by SDS-PAGE, and transferred to PVDF membranes (Millipore, Billerica, MA, USA). The membranes were blocked with a TBS-T buffer containing 5% nonfat skim milk at room temperature for 1 h. The membranes were incubated with primary antibodies overnight at 4 °C: anti-pCREB (Ser133) (#9196, Cell Signaling Technology, Berkeley, CA, USA), anti-CREB (#9197, Cell Signaling Technology, Berkeley, CA, USA), anti-GAPDH (sc-25778, Santa Cruz Biotechnology, Santa Cruz, CA, USA), anti-tyrosinase (T311) (sc-20035, Santa Cruz Biotechnology, Santa Cruz, CA, USA), anti-TYRP1 (G-9) (sc-166857, Santa Cruz Biotechnology, Santa Cruz, CA, USA), and anti-TYRP2 (C-9) (sc-74439, Santa Cruz Biotechnology, Santa Cruz, CA, USA). After the membranes were washed with the TBS-T buffer, they were incubated with HRP-conjugated secondary antibodies at room temperature for 1 h. The membrane-bound proteins were visualized using a Pierce^TM^ ECL Western Blotting Substrate (Thermo Fisher Scientific, Rockford, IL, USA). To detect the MITF and histone 3 proteins, the total nuclear proteins were prepared with NE-PER Nuclear and Cytoplasmic Extraction Reagents (Thermo Fisher Scientific, Rockford, IL, USA) according to the manufacturer’s instructions. The protein concentration was measured using a Bio-Rad Protein Assay Kit (Bio-Rad, Hercules, CA, USA), and then the experimental procedure just described was performed. The primary antibodies used were anti-MITF (sc-515925, Santa Cruz Biotechnology, Santa Cruz, CA, USA) and anti-histone H3 (D1H2) (#4499, Cell Signaling Technology, Berkeley, CA, USA).

### 4.8. Statistical Analysis

Statistical analyses were conducted using the GraphPad Prism 9 software (San Diego, CA, USA). All the data obtained from the cell toxicity assay, qRT-PCR, melanin synthesis assay, and Western blot analysis are expressed as the means ± standard deviation (SD). The differences between the cells from different conditions obtained from the cell toxicity assay, melanin synthesis assay, qRT-PCR, and Western blot analysis were analyzed with one-way ANOVA followed by the HSD test; *p* < 0.05 was considered statistically significant.

## 5. Conclusions

This study demonstrated that Ech A inhibits melanogenesis and its underlying mechanism, indicating it as a potential skin-whitening agent for functional cosmetics.

## Figures and Tables

**Table 1:** The primers used in qRT-PCR.

Gene	Forward (5′-3′)	Reverse (5′-3′)	Amplicon Size (bp)
*Gapdh*	AGGTCGGTGTGAACGGATTTG	TGTAGACCATGTAGTTGAGGTCA	123
*Mitf*	ACTTTCCCTTATCCCATCCACC	TGAGATCCAGAGTTGTCGTACA	143
*Tyrp1*	CCCCTAGCCTATATCTCCCTTTT	TACCATCGTGGGGATAATGGC	229
*Tyrp2*	TTCTGCTGGGTTGTCTGGG	CACAGATGTTGGTTGCCTCG	135
*Tyr*	CTCTGGGCTTAGCAGTAGGC	GCAAGCTGTGGTAGTCGTCT	107

## Data Availability

Not applicable.

## Supplementary Materials

The following supporting information can be downloaded at https://www.mdpi.com/article/10.3390/md20090555/s1, Figure S1: Cytotoxicity of B16F10 cells in response to kojic acid; Table S1: Inhibitory effects of arbutin on the activity of tyrosinase and oxidation of L-DOPA.
