# Supplementary material for: Echinochrome A Inhibits Melanogenesis in B16F10 Cells by Downregulating CREB Signaling
Source: Mar Drugs. 2022 Aug 29;20(9):555. doi: 10.3390/md20090555 (PMC9502928; doi:10.3390/md20090555)
Supplement: Supplementary file 1 [file marinedrugs-20-00555-s001.zip › Figure S1.pptx]

## Slide 1
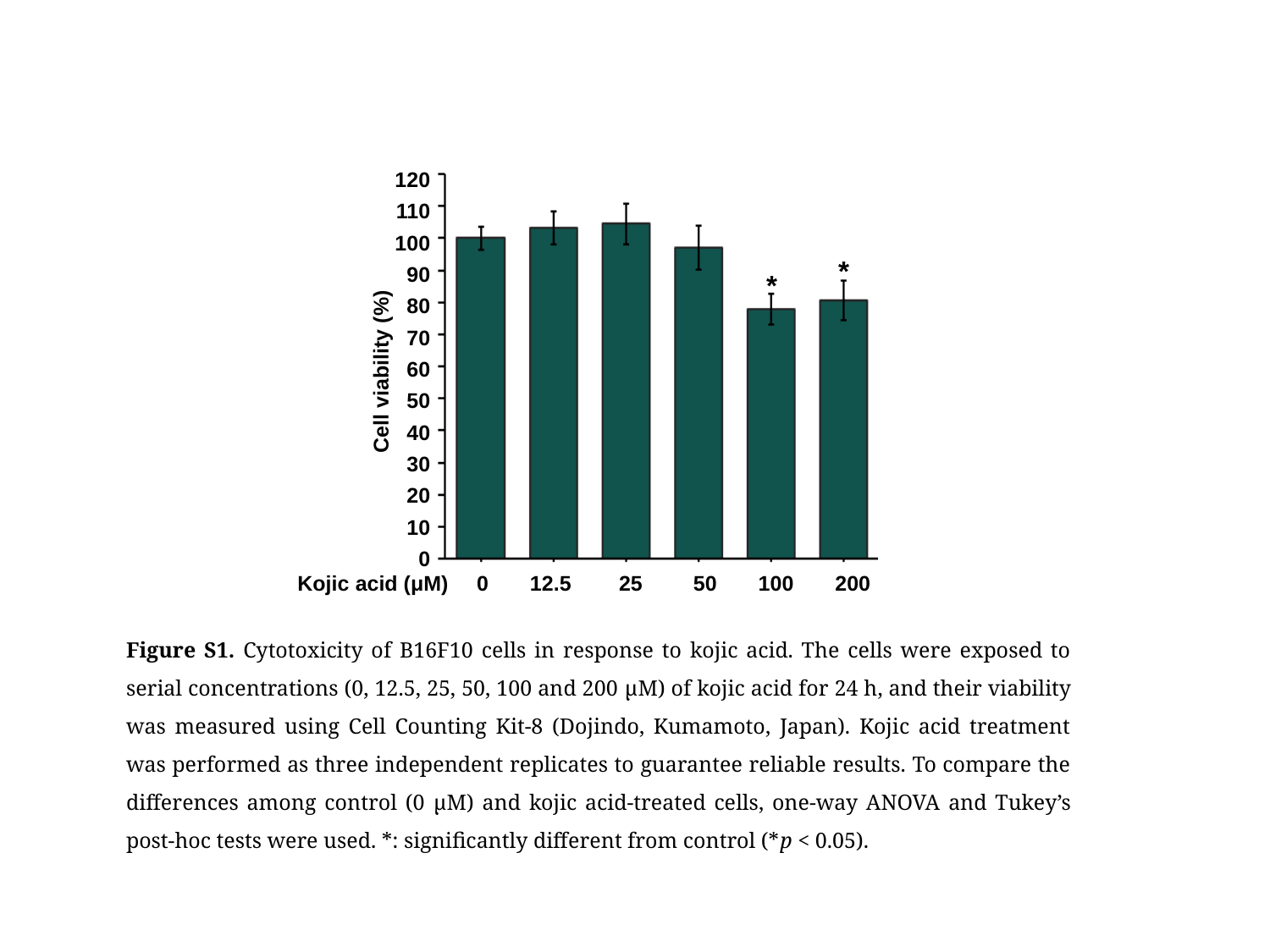

120
110
100
90
80
70
60
50
40
30
20
10
0
*
*
Cell viability (%)
Kojic acid (μM)
0 12.5 25 50 100 200
Figure S1. Cytotoxicity of B16F10 cells in response to kojic acid. The cells were exposed to serial concentrations (0, 12.5, 25, 50, 100 and 200 μM) of kojic acid for 24 h, and their viability was measured using Cell Counting Kit-8 (Dojindo, Kumamoto, Japan). Kojic acid treatment was performed as three independent replicates to guarantee reliable results. To compare the differences among control (0 μM) and kojic acid-treated cells, one-way ANOVA and Tukey’s post-hoc tests were used. *: significantly different from control (*p < 0.05).
