# Supplementary material for: Echinochrome A Inhibits Melanogenesis in B16F10 Cells by Downregulating CREB Signaling
Source: Mar Drugs. 2022 Aug 29;20(9):555. doi: 10.3390/md20090555 (PMC9502928; doi:10.3390/md20090555)
Supplement: Supplementary file 1 [file marinedrugs-20-00555-s001.zip › Table S1.pdf]

Table S1. Inhibitory effects of arbutin on the activity of tyrosinase and oxidation of L-DOPA.

**Arbutin (μM)**	**Inhibition rate of tyrosinase activity (%)**	**Inhibition rate of L-DOPA oxidation (%)**
0	0	0
72	44.25 ± 2.39	-
360	74.81 ± 7.54	-
720	70.74 ± 4.76	-
900	73.88 ± 5.39	46.28 ± 0.40
1,835	83.57 ± 6.74	50.80 ± 4.11
3,670	-[a]	63.01 ± 6.81
7,340	-	73.34 ± 5.36
11,010	-	73.21 ± 1.77
14,680	-	88.22 ± 2.25

[a]: No measurement. IC_{50} concentration of arbutin to inhibit tyrosinase activity: 126 ± 23.11 μM. IC_{50} concentration of arbutin to inhibit L-DOPA oxidation: 3,279.74 ± 17.14 μM.
